# Supplementary material for: Synovial Fluid Extracellular Vesicles from Patients with Severe Osteoarthritis Differentially Promote a Pro-Catabolic, Inflammatory Chondrocyte Phenotype
Source: Biomolecules. 2025 Jun 6;15(6):829. doi: 10.3390/biom15060829 (PMC12190261; doi:10.3390/biom15060829)
Supplement: Supplementary file 1 [file biomolecules-15-00829-s001.zip › Supplementary Figure S1 Workflow schematic.pptx]

## Slide 1
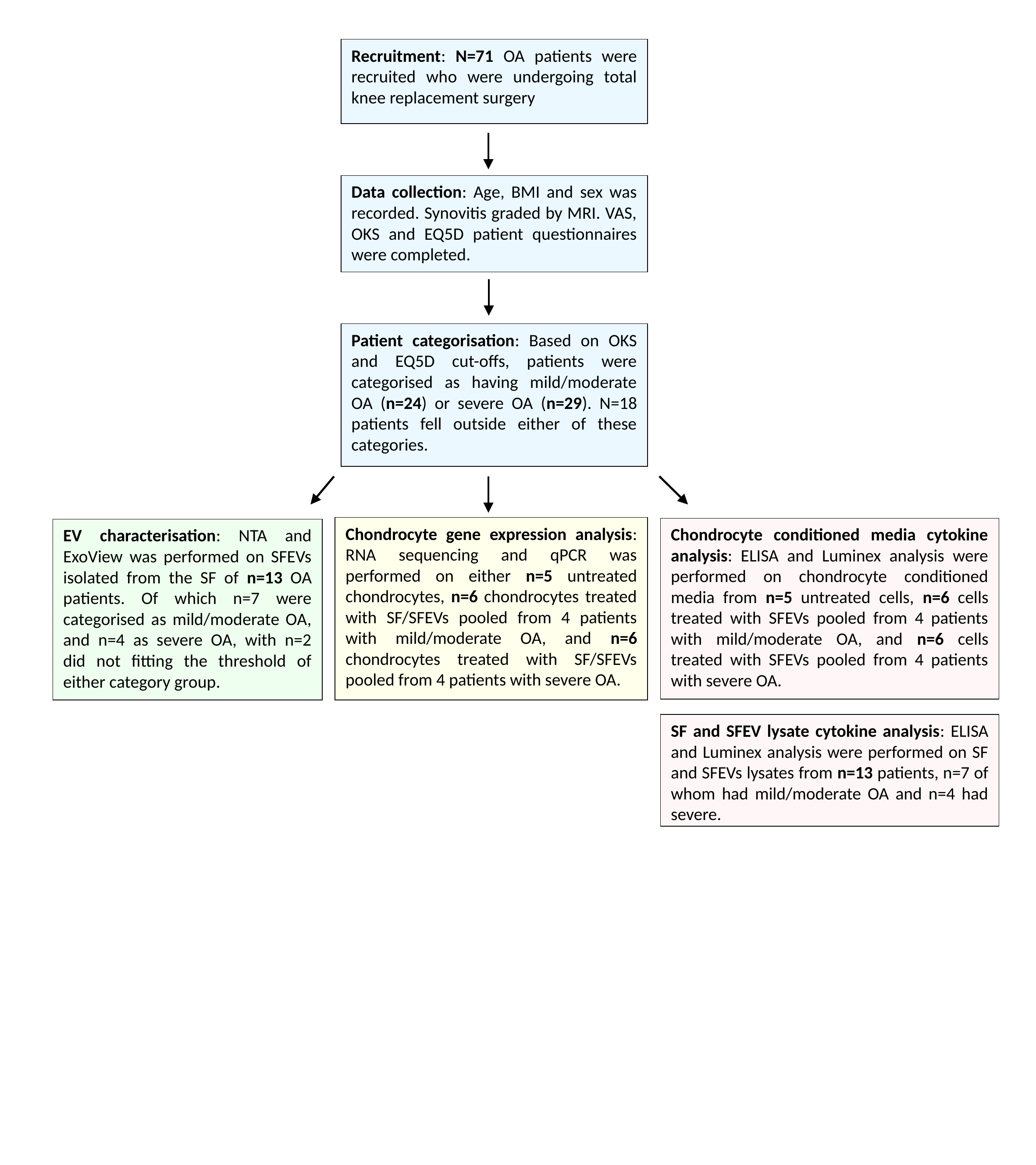

Recruitment: N=71 OA patients were recruited who were undergoing total knee replacement surgery
Data collection: Age, BMI and sex was recorded. Synovitis graded by MRI. VAS, OKS and EQ5D patient questionnaires were completed.
Patient categorisation: Based on OKS and EQ5D cut-offs, patients were categorised as having mild/moderate OA (n=24) or severe OA (n=29). N=18 patients fell outside either of these categories.
Chondrocyte gene expression analysis: RNA sequencing and qPCR was performed on either n=5 untreated chondrocytes, n=6 chondrocytes treated with SF/SFEVs pooled from 4 patients with mild/moderate OA, and n=6 chondrocytes treated with SF/SFEVs pooled from 4 patients with severe OA.
Chondrocyte conditioned media cytokine analysis: ELISA and Luminex analysis were performed on chondrocyte conditioned media from n=5 untreated cells, n=6 cells treated with SFEVs pooled from 4 patients with mild/moderate OA, and n=6 cells treated with SFEVs pooled from 4 patients with severe OA.
EV characterisation: NTA and ExoView was performed on SFEVs isolated from the SF of n=13 OA patients. Of which n=7 were categorised as mild/moderate OA, and n=4 as severe OA, with n=2 did not fitting the threshold of either category group.
SF and SFEV lysate cytokine analysis: ELISA and Luminex analysis were performed on SF and SFEVs lysates from n=13 patients, n=7 of whom had mild/moderate OA and n=4 had severe.
