# Supplementary figures and images for: Synovial Fluid Extracellular Vesicles from Patients with Severe Osteoarthritis Differentially Promote a Pro-Catabolic, Inflammatory Chondrocyte Phenotype
Source: Biomolecules. 2025 Jun 6;15(6):829. doi: 10.3390/biom15060829 (PMC12190261; doi:10.3390/biom15060829)

## Slide 1
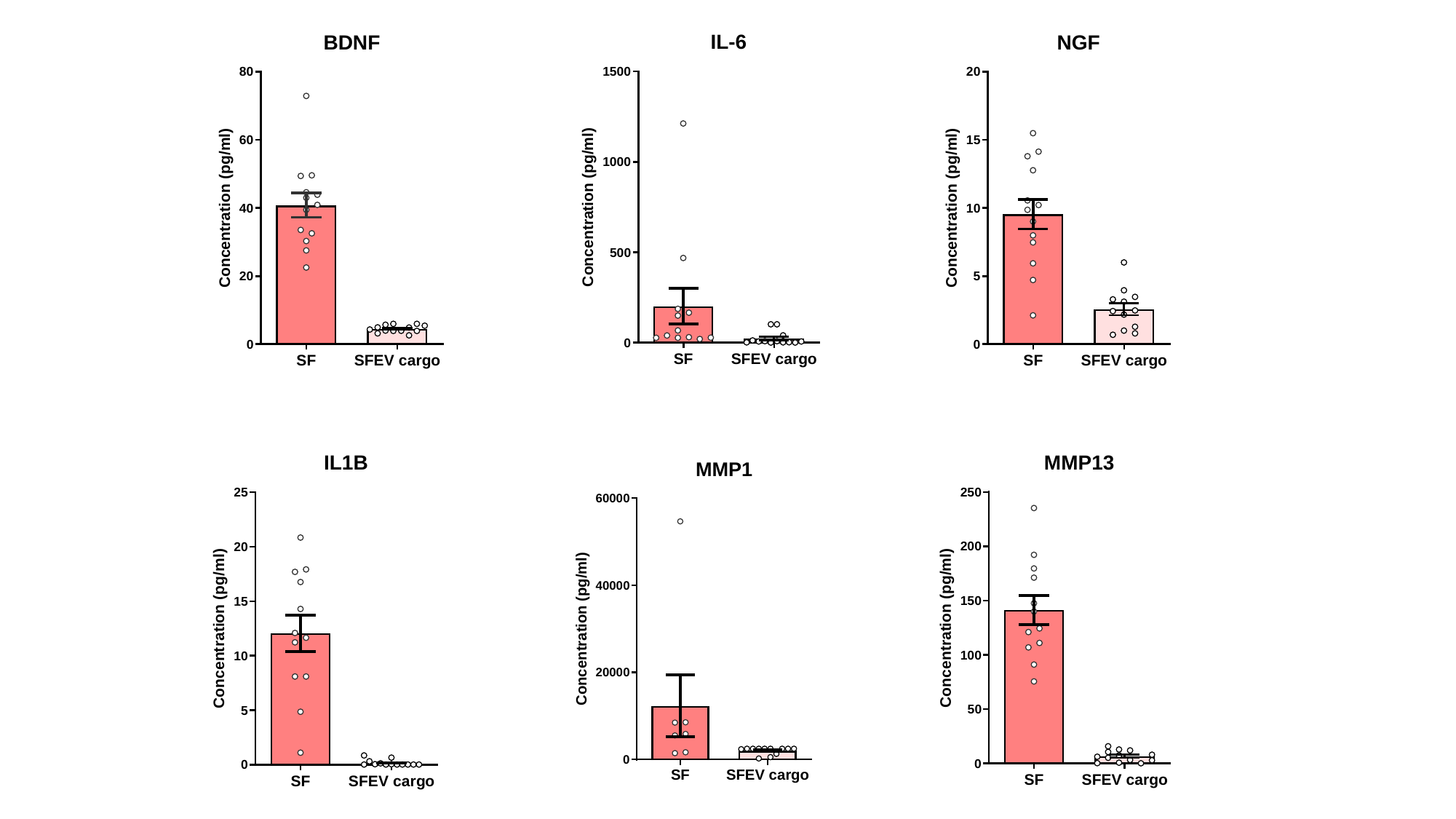

Supplement: Supplementary file 1 [file biomolecules-15-00829-s001.zip › Supplementary Figure S2.pptx]
